# Elevated body temperature is associated with depressive symptoms: results from the TemPredict Study

**DOI:** 10.1038/s41598-024-51567-w

**Published:** 2024-02-05

**Authors:** Ashley E. Mason, Patrick Kasl, Severine Soltani, Abigail Green, Wendy Hartogensis, Stephan Dilchert, Anoushka Chowdhary, Leena S. Pandya, Chelsea J. Siwik, Simmie L. Foster, Maren Nyer, Christopher A. Lowry, Charles L. Raison, Frederick M. Hecht, Benjamin L. Smarr

**Affiliations:** 1 Osher Center for Integrative Health, University of California San Francisco, San Francisco, CA USA; 2 Shu Chien-Gene Lay Department of Bioengineering, University of California San Diego, San Diego, CA USA; 3 Neurosciences Graduate Program, University of California San Diego, San Diego, CA USA; 4 Department of Management, Zicklin School of Business, Baruch College, The City University of New York, New York, NY USA; 5 Department of Psychology, The University of Arizona, Tucson, AZ USA; 6 Department of Wellness and Preventative Medicine, Cleveland Clinic, Cleveland, OH USA; 7 Depression Clinical and Research Program, Massachusetts General Hospital, Boston, MA USA; 8 Department of Psychiatry, Harvard Medical School, Boston, MA USA; 9 Department of Integrative Physiology, University of Colorado Boulder, Boulder, CO USA; 10 Department of Psychiatry, School of Medicine and Public Health, University of Wisconsin-Madison, Madison, WI USA; 11 Halıcıoğlu Data Science Institute, University of California San Diego, San Diego, CA USA

**Keywords:** Psychology, Human behaviour, Diagnostic markers

## Abstract

Correlations between altered body temperature and depression have been reported in small samples; greater confidence in these associations would provide a rationale for further examining potential mechanisms of depression related to body temperature regulation. We sought to test the hypotheses that greater depression symptom severity is associated with (1) higher body temperature, (2) smaller differences between body temperature when awake versus asleep, and (3) lower diurnal body temperature amplitude. Data collected included both self-reported body temperature (using standard thermometers), wearable sensor-assessed distal body temperature (using an off-the-shelf wearable sensor that collected minute-level physiological data), and self-reported depressive symptoms from > 20,000 participants over the course of ~ 7 months as part of the TemPredict Study. Higher self-reported and wearable sensor-assessed body temperatures when awake were associated with greater depression symptom severity. Lower diurnal body temperature amplitude, computed using wearable sensor-assessed distal body temperature data, tended to be associated with greater depression symptom severity, though this association did not achieve statistical significance. These findings, drawn from a large sample, replicate and expand upon prior data pointing to body temperature alterations as potentially relevant factors in depression etiology and may hold implications for development of novel approaches to the treatment of major depressive disorder.

## Introduction

Depression has become a health crisis of epidemic proportions^1^. Globally, the prevalence of major depressive disorder (MDD) has risen over the last several generations in countries across the world^2^. The last decade has seen a particularly significant increase in depression in the United States, with prevalence rates increasing by 33% between 2013 and 2016, with the largest increase among youth and young adults^3,4^. This is particularly concerning as the disease course is most likely to be malignant, and the costs of depression in terms of lost opportunities across a lifetime are likely to be highest in youth and young adulthood^4^. Rates of antidepressant use have increased substantially in most Western countries during the same time period^5^, and currently available pharmacologic agents have significant limitations in efficacy^6–8^. Taken together, these patterns highlight the urgent need to identify and implement new treatments for depression.

To develop novel treatments for depression it is important to identify mechanisms that contribute to the development and/or maintenance of depressive symptoms and that may be amenable to intervention. Although depression is both biologically and behaviorally heterogeneous^9,10^, an important first step in treatment development is often to identify physiologic signatures among individuals with MDD that are not present among those without MDD. Although no single biological or behavioral abnormality will characterize all individuals with MDD, the identification of an abnormality associated with MDD may open the door to identifying a relatively biologically homogeneous subgroup that demonstrates a larger treatment response to interventions that target the specific abnormality^11^.

One physiologic characteristic that may hold potential as a therapeutic target is thermoregulatory dysregulation^12^, which is among the most widely reported circadian biological abnormalities in affective disorders, including MDD. This has been observed in the form of elevated body temperature, particularly at night^13–16^, when thermoregulatory cooling responses are critical for sleep onset and quality^17,18^. Such temperature elevations have also been reported during the day^19,20^. Notably, some data suggest that these aberrant temperature elevations improve upon clinical recovery in MDD^21,22^. Data have shown that circadian amplitude is blunted in depression^23^ and that smaller differences between average body temperature during time awake (typically during the daytime) and during time asleep (typically during the nighttime) are associated with greater depressive symptoms^21^. Additionally, data have shown that individuals with depression have lower circadian body temperature amplitudes that increase upon clinical recovery^13,21^. These alterations are notable in light of the narrow range of human body temperature^24,25^.

Although suggestive, these results derive from small-scale studies conducted with limited sample sizes (< 300) and in controlled settings^13–15,19,20^. Thus, an important step toward understanding the association between thermoregulatory dysfunction and depressive symptoms is to establish this association outside of clinical laboratory settings and in larger samples. Establishing a more definitive association between measures of body temperature and depression would set the stage for further explorations of depression treatment modalities that target the thermoregulatory system. To accomplish this, the current analyses tested associations between monthly depression symptom scores and (1) self-collected body temperature submitted to the research team via online surveys and (2) wearable sensor device-collected distal body temperature automatically transmitted to the research team over the internet, in a large international sample (*N* > 20,000) of adults who participated in an online study focused on COVID-19 detection beginning in March of 2020. Initial analyses used self-reported body temperature, which participants collected using personal thermometers (e.g., oral temperature) and reported via daily surveys. Further analyses used minute-level distal body temperature data collected using an off-the-shelf wearable sensor device. In total, using wearable sensor-assessed distal body temperature, we analyzed four body temperature metrics examined in prior research^13–16,19–21^: (1) distal body temperature while awake; (2) distal body temperature while asleep; (3) the difference between the average distal body temperatures while asleep and awake; and (4) the diurnal distal body temperature amplitude. Based on prior studies with small samples and our prior theoretical work^12–16,19–21^, we hypothesized that higher self-reported body temperature and wearable sensor-assessed distal body temperatures, lower diurnal distal body temperature amplitude, and smaller differences between awake and asleep distal body temperature, would be associated with higher levels of depressive symptoms.

## Results

### Study overview

The TemPredict Study, initiated in March of 2020, sought to assess whether off-the-shelf wearable sensor devices collect data that could be used to screen large numbers of individuals for the early stages of SARS-CoV-2 infection^26,27^. The primary findings included that physiological data allowed for prediction of a COVID-19 infection 2.75 days prior to diagnosis. All participants wore a commercially available off-the-shelf wearable sensor device, the Oura Ring (Oura Health, Oulu, Finland) that collected distal body temperature (one value per minute while worn on the finger) and paired with a smartphone app. Participants also completed daily surveys that asked for a self-collected body temperature measurement (assessed with a handheld thermometer; not assessed using the wearable device), a baseline survey that collected demographic data, and monthly surveys that included mental health assessments.

The self-reported body temperature analytic sample included 20,880 individuals (Supplementary Fig. S1). The mean age (standard deviation [SD]) was 46.9 (12.6) years; 53% male; 47% female. For adjusted analyses, we excluded participants who reported their biological sex as ‘other’ and participants whose biological sex was missing (*n* = 17). We first computed each participant’s average T-score across a maximum of seven monthly PROMIS depression assessments. We then computed the average of all participants’ mean T-scores (SD); this value was 51.49 (7.37), which is within normal limits (WNL). On average, participants completed 3.6 of 7 possible PROMIS depression assessments and self-reported a total of 559,664 body temperature assessments (mean of 27 daily temperature reports per participant).

The wearable sensor-assessed body temperature analytic sample included 21,064 individuals (Supplementary Fig. S2). The mean age (SD) was 46.5 (12.1) years; 56% male; 44% female. The average T-score across a maximum of seven monthly PROMIS depression assessments in this sample was 50.94 (7.26), which is also WNL. On average, participants completed 4.0 of 7 possible PROMIS depression assessments; we used 2 weeks of wearable sensor device distal body temperature data prior to each completed PROMIS assessment for analyses. Participants had an average of 35.5 days of distal body temperature data with at least (1) 4 h of distal body temperature data during the asleep period and (2) at least 4 h of distal body temperature data during the awake period for each 24-h period available within our timeframe of analyses. Both analytic samples (which we drew from the same participant pool) included a geographically diverse set of individuals from 106 different countries.

### Self-reported body temperature

#### Depression symptom frequencies

Of the 20,880 participants with self-reported temperature data, 13,595 had an average depression symptom severity WNL (T-score < 55), 4527 had an average depression symptom severity within the mild range (T-score ≥ 55 and < 60), 2666 had an average depression symptom severity within the moderate range (T-score ≥ 60 and < 70), and 92 had an average depression symptom severity within the severe range (T-score ≥ 70) of the PROMIS depression assessment^28^. The average depression symptom severity score across participants’ average scores was 51.5 (the average within-person SD = 2.5), and the average range between participants’ lowest to highest scores was 5.2 (SD = 5.5), and the median was 3.9 (interquartile range [IQR] = 0–8.3).

#### Graphical depictions of self-report temperature data

**Figure 1:**
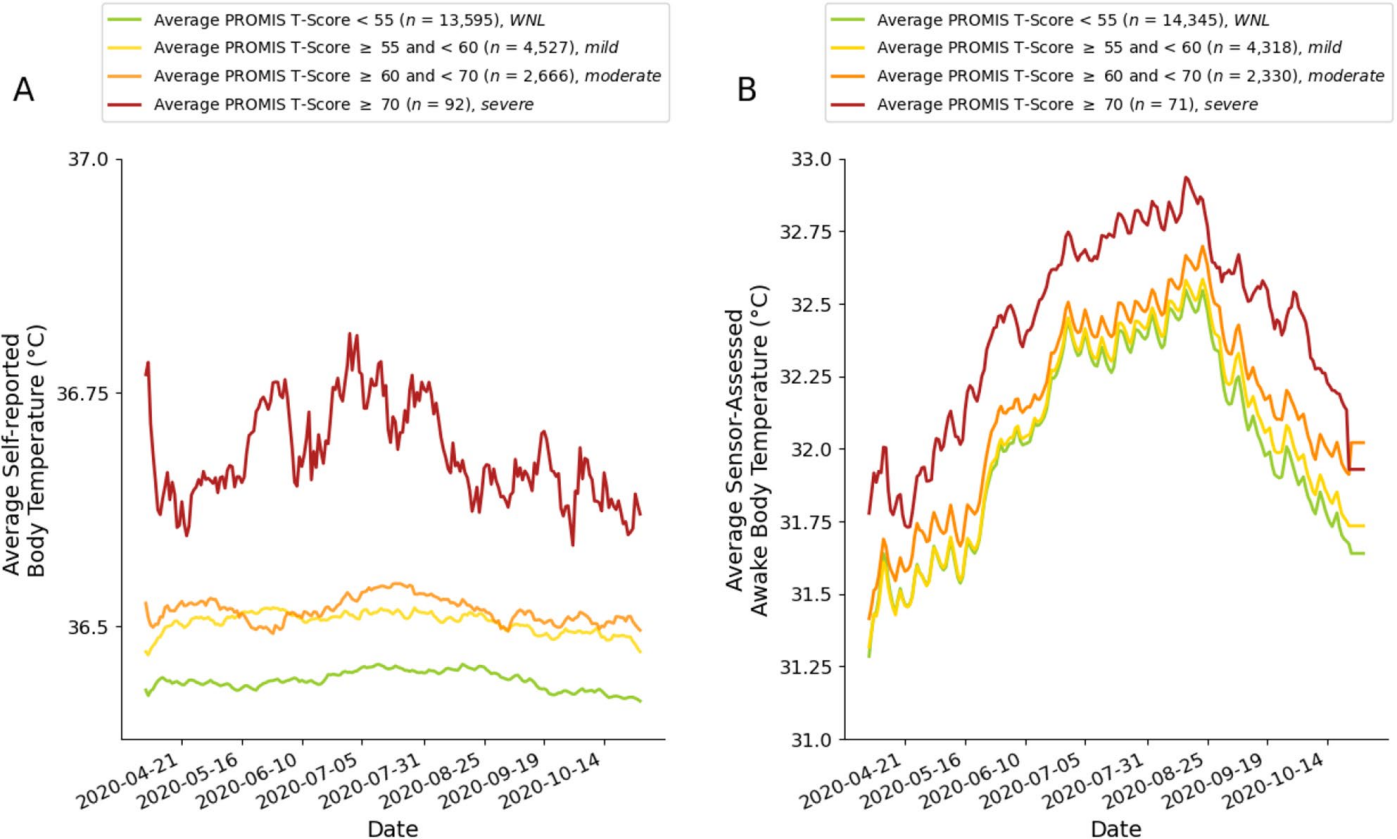
Average self-reported body temperature (**A**) and average wearable sensor-assessed distal body temperature (**B**) plotted by PROMIS depression symptom T-score categories. Figure panels show that individuals with PROMIS depression symptom T-scores within normal limits (WNL; green) have the lowest average self-reported and wearable sensor-assessed distal body temperatures, with increasing average self-reported and wearable sensor-assessed distal body temperatures among individuals in the mild (yellow), moderate (orange), and severe (red) PROMIS depression symptom T-score categories. *Note*. We took the self-reported body temperature data from each calendar day (Panel **A**; most frequently reported by participants in the morning) and the average of any available wearable sensor-assessed distal body temperature data from each calendar day (during awake time; Panel **B**) and plotted those average values for the different depression groups. We smoothed average self-reported and wearable sensor-assessed body temperatures (°C) using an exponentially weighted moving average with a 7-day window before and after each timepoint as a function of the average NIH PROMIS Adult Health Profile instrument for depression (Form 4a) score. See Supplementary Fig. S3 for unsmoothed figure panels.

**Figure 2:**
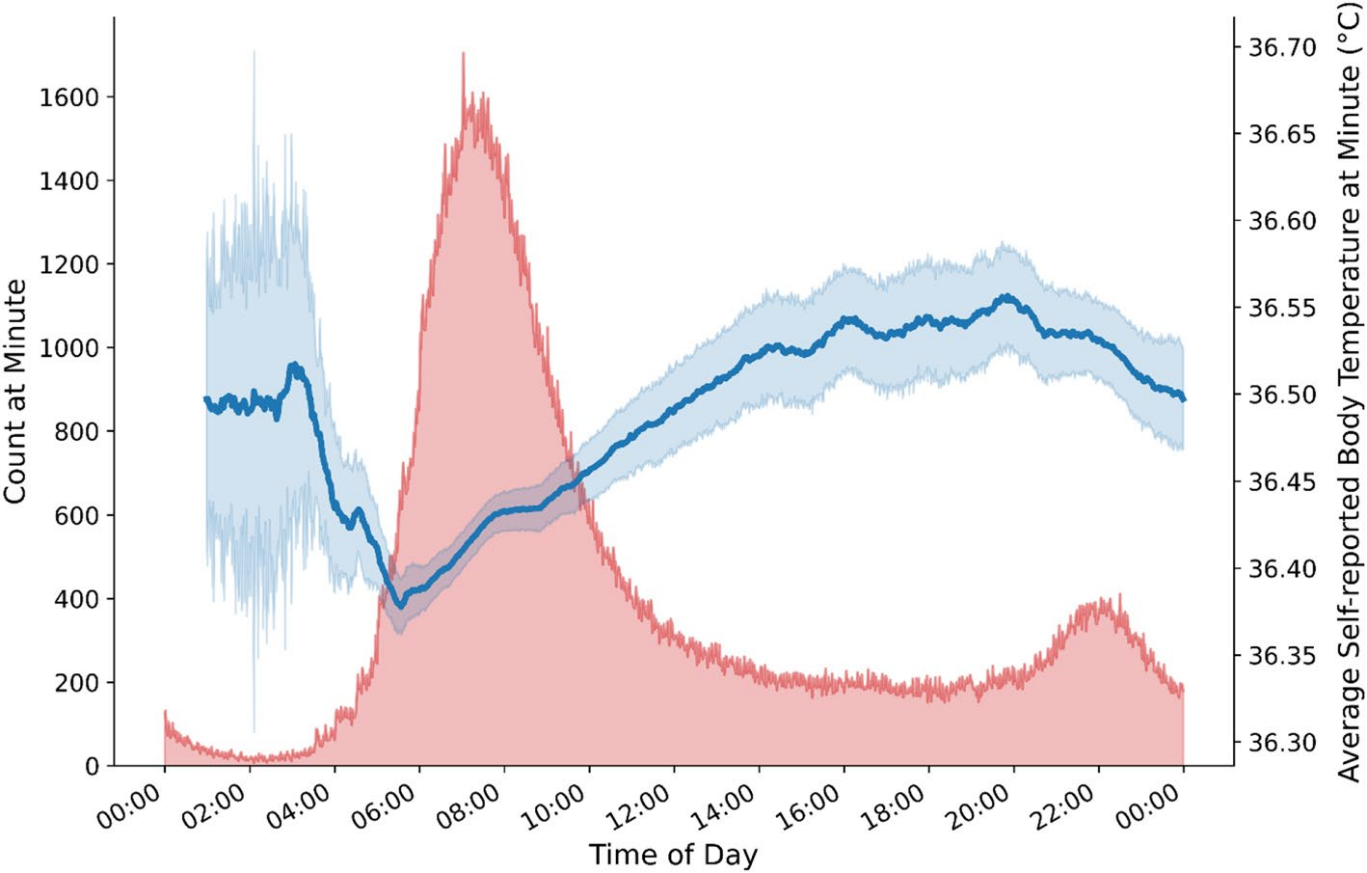
Average self-reported body temperature by time-of-day. Figure depicts expected diurnal pattern of lowest self-reported body temperatures reported in the early morning hours and higher self-reported body temperatures during daytime hours. *Note.* Blue line depicts average self-reported body temperature (right Y axis) by time of day; blue shading indicates standard error of the mean. Red shading indicates number of responses (left Y axis) provided at each minute (X axis).

Figure 1 illustrates the differences in average self-reported body temperature across participants with average PROMIS depression symptom T-scores WNL, and in the mild, moderate, and severe ranges. The self-reported, time-stamped body temperature survey data (adjusted for local time zone) revealed that participants’ self-reported temperatures followed expected diurnal patterns (Fig. 2), with lower body temperatures in the early morning and rising body temperatures during daytime hours that fell in the evening hours. This is consistent with the interpretation that participants self-collected their body temperature around the same time that they reported their body temperature to the study. As shown, participants tended to self-report their body temperature more often in the morning than in the evening.

#### Linear regression models

We used linear regression models to assess whether the average daily self-reported body temperature recorded over seven months was correlated with the average PROMIS depression T-score across seven monthly assessments from the same period. We found that these were positively correlated (greater body temperature associated with greater depression T-score) in an unadjusted model (*b* = 1.6661; 95% CI [1.4128,1.9195]; *p* = 7.1 × 10^–38^) and in a model adjusted for age, biological sex, and self-reported body temperature survey time stamp (*b* = 0.8595; 95% CI [0.6118,1.1071]; *p* = 1.1 × 10^–11^], Table 1). We calculated E-values as sensitivity analyses. E-values for these linear models ranged from 1.38 to 1.60, and in all cases were larger than observed effects of body temperature, age, or biological sex on depression (Table 1), meaning that it is unlikely that unaddressed or unmeasured confounders had effects that better explained the reported (observed) effects. Standardized adjusted regression analyses showed that body temperature accounted for unique variance in PROMIS depression T-scores beyond the known variance accounted for by biological sex and age (Supplementary Table S1).

**Table 1:** Unadjusted and adjusted linear models regressing average PROMIS depression symptom T-scores onto average self-reported body temperature.

Model	Model parameter	*b*	SE	*p*	95% CI (LB, UB)	E-value for point estimate (E-value for 95% CI)
Unadjusted (*n* = 20,880) *r*^*2*^ = 0.008 *p* = 7.1 × 10^–38^	Intercept	51.488	0.051		[51.388, 51.588]	1.60 (1.53)
	Self-report body temperature	1.666	0.129	7.1 × 10^–38^	[1.413, 1.920]	
Adjusted (*n* = 20,863) *r*^2^ = 0.078 *p* = 2.3 × 10^–128^	Intercept	52.940	0.0764		[52.790, 53.089]	1.38 (1.30)
	Self-report body temperature	0.859	0.126	1.1 × 10^–11^	[0.612, 1.107]	
	Age	− 0.103	0.004	9.3 × 10^–146^	[− 0.111, − 0.096]	
	Biological sex	− 2.741	0.099	1.8 × 10^–165^	[− 2.935, − 2.547]	
	Time of day (B1)	− 0.526	0.117	7.5 × 10^–6^	[− 0.756, − 0.296]	
	Time of day (B2)	− 0.774	0.095	4.6 × 10^–16^	[− 0.961, − 0.588]	

*Note*. Models predict depression symptom T-scores assessed using the Patient-Reported Outcomes Measurement Information System (PROMIS), Adult Health instrument for depression (Form 4a), modified to reflect a 1-month timeframe^63^. We used survey timestamps to compute the cosine (Time of Day B1) and sine (Time of Day B2) of 2*pi*t for each self-reported body temperature measurement, where “t” was the decimal proportion of the day during which the daily survey was completed (see “Methods”). CI, confidence interval; LB, lower bound; UB, upper bound. Mean self-reported body temperatures were centered around the grand mean of 36.54, such that the intercept represents the mean PROMIS depression T-score for participants with average self-reported body temperatures at the analytic sample mean. Age was centered around the overall analytic sample mean of 46.94.

#### Logistic regression models

**Figure 3:**
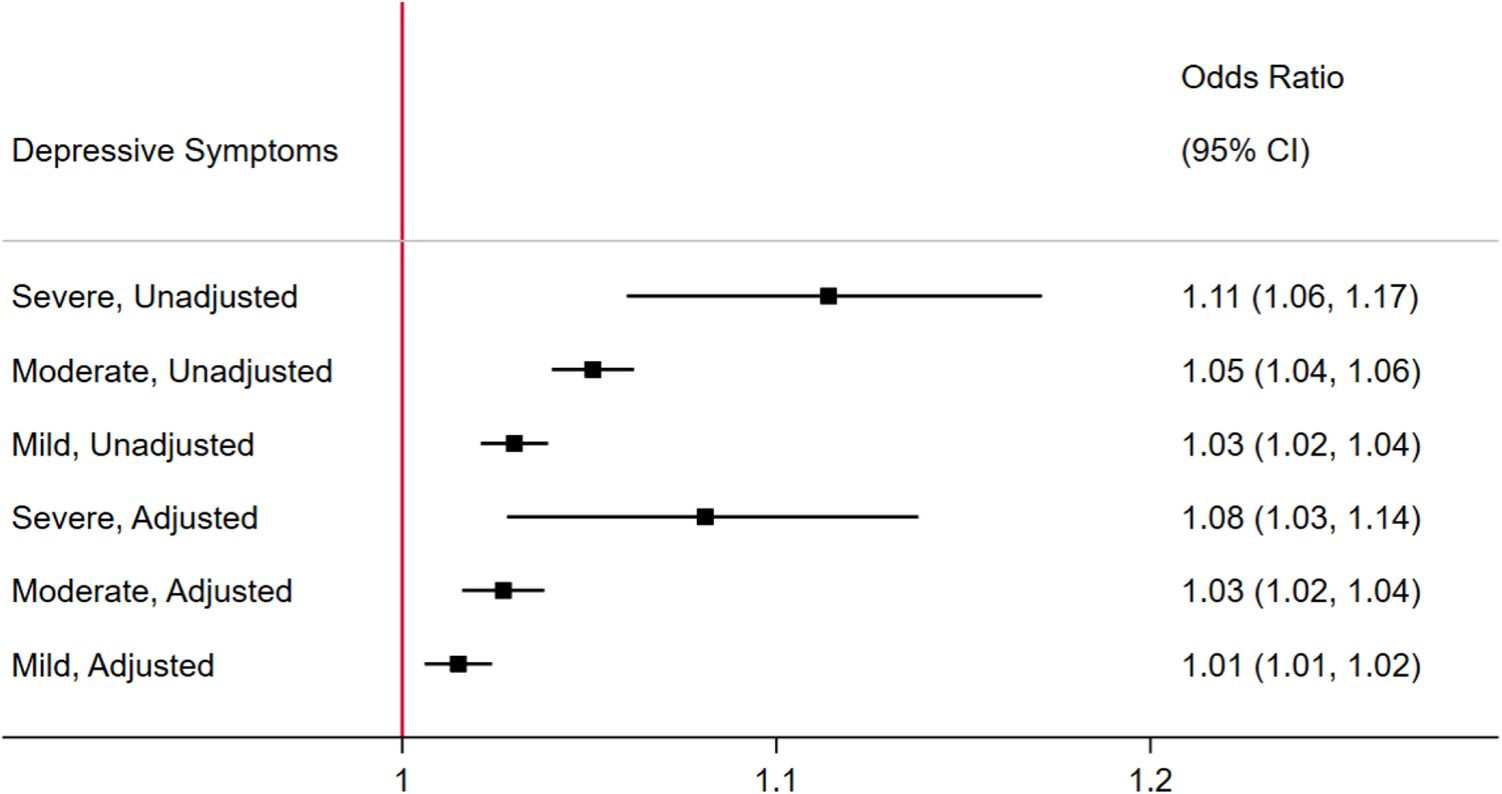
Forest plot depicting odds ratios from logistic regression models. Adjusted and unadjusted models predict PROMIS depression T-score categories (mild, moderate, severe) vs. depressive symptoms within normal limits (WNL) from self-reported body temperature (scaled per 0.1 °C).

We next tested whether greater self-reported body temperature was associated with increased odds of having depression using separate logistic regression models for each category of depression (mild, moderate, and severe). In unadjusted analyses (Table 2), the odds ratio for having average PROMIS depression T-scores within the mild range vs. WNL were significantly increased with each 0.1 °C increase in average body temperature (OR: 1.030; 95% CI [1.021,1.039]; *p* = 1.4 × 10^–11^). Similarly, there were increased (but progressively greater) odds of having (with each 0.1 °C increase in body temperature) PROMIS depression T-scores within the moderate range (OR = 1.051 [95% CI, 1.040, 1.062], *p* = 2.3 × 10^–20^) and of having PROMIS depression T-scores within the severe range (OR: 1.114; 95% CI [1.060, 1.171], *p* = 2.1 × 10^–5^) relative to PROMIS depression T-scores WNL (Fig. 3). Similar regression analyses adjusting for covariates attenuated these differences somewhat (mild vs. WNL; OR: 1.015; 95% CI [1.006, 1.024], *p* = 8.7 × 10^–4^; moderate vs. WNL OR: 1.027 95% CI [1.016, 1.038], *p* = 1.8 × 10^–6^; severe vs. WNL: OR = 1.081; 95% CI [1.028, 1.138], *p* = 0.0026; Table 3). E-values for these logistic models ranged from 1.09 to 1.30, and in all cases were larger than observed effects of self-reported body temperature, age, or biological sex, on depression (Tables 2 and 3).

**Table 2:** Unadjusted logistic models regressing PROMIS depression symptom T-score categories (mild, moderate, severe, with T-scores within normal limits [WNL] as the reference category) onto average self-reported body temperature (scaled per 0.1 °C).

Model	Model Parameter	OR	SE	*p*	95% CI [LB, UB]	E-Value of point estimate (E-Value of 95% CI)
Severe Depressive Symptoms vs. WNL 13,687 observations Pseudo *r*^2^ = 0.0157	Intercept	0.006	0.001		[0.005, 0.008]	1.30 (1.20)
	Self-report body temperature	1.114	0.028	2.1 × 10^–5^	[1.060, 1.171]	
Moderate Depressive Symptoms vs. WNL 16,261 observations Pseudo* r*^2^ = 0.0059	Intercept	0.194	0.004		[0.186, 0.203]	1.19 (1.16)
	Self-report body temperature	1.051	0.006	2.3 × 10^–20^	[1.040, 1.062]	
Mild Depressive Symptoms vs. WNL 18,122 observations Pseudo* r*^2^ = 0.0022	Intercept	0.333	0.006		[0.322, 0.344]	1.14 (1.11)
	Self-report body temperature	1.030	0.005	1.4 × 10^–11^	[1.021, 1.039]	

*Note*. CI, confidence interval; LB, lower bound; UB, upper bound.

**Table 3:** Adjusted logistic models regressing PROMIS depression symptom T-score categories (mild, moderate, severe, with T-scores within normal limits [WNL] as the reference category) onto average self-reported body temperature (scaled per 0.1 °C).

Model	Model parameter	OR	SE	*p*	95% CI [LB, UB]	E-Value of point estimate (E-Value of 95% CI)
Severe Depressive Symptoms vs. WNL 13,676 observations Pseudo* r*^2^ = 0.0742	Intercept	0.007	0.001		[0.005, 0.010]	1.24 (1.13)
	Self-report body temperature	1.081	0.028	0.0026	[1.028, 1.138]	
	Age	0.941	0.009	8.1 × 10^–10^	[0.922, 0.959]	
	Biological sex	0.580	0.124	0.011	[0.381, 0.883]	
	Time of day (B1)	0.894	0.208	0.63	[0.567, 1.410]	
	Time of day (B2)	0.557	0.113	0.0039	[0.375, 0.829]	
Moderate Depressive Symptoms vs. WNL 16,246 observations Pseudo* r*^2^ = 0.0582	Intercept	0.261	0.008		[0.245, 0.278]	1.13 (1.10)
	Self-report body temperature	1.027	0.006	1.8 × 10^–06^	[1.016, 1.038]	
	Age	0.965	0.002	3.2 × 10^–81^	[0.961, 0.968]	
	Biological sex	0.477	0.021	3.2 × 10^–62^	[0.437, 0.520]	
	Time of day (B1)	0.786	0.040	2.7 × 10^–6^	[0.710, 0.869]	
	Time of day (B2)	0.762	0.032	1.1 × 10^–10^	[0.701, 0.827]	
Mild Depressive Symptoms vs. WNL 18,109 observations Pseudo* r*^2^ = 0.0257	Intercept	0.431	0.011		[0.410, 0.454]	1.09 (1.06)
	Self-report body temperature	1.015	0.005	8.7 × 10^–4^	[1.006, 1.024]	
	Age	0.979	0.001	2.9 × 10^–47^	[0.976, 0.982]	
	Biological sex	0.591	0.021	1.6 × 10^–50^	[0.552, 0.633]	
	Time of day (B1)	0.869	0.036	7.0 × 10^–4^	[0.801, 0.942]	
	Time of day (B2)	0.855	0.029	3.2 × 10^–6^	[0.800, 0.913]	

*Note*. See Table 2 note.

#### Receiver operating characteristics (ROC) curve analyses

**Figure 4:**
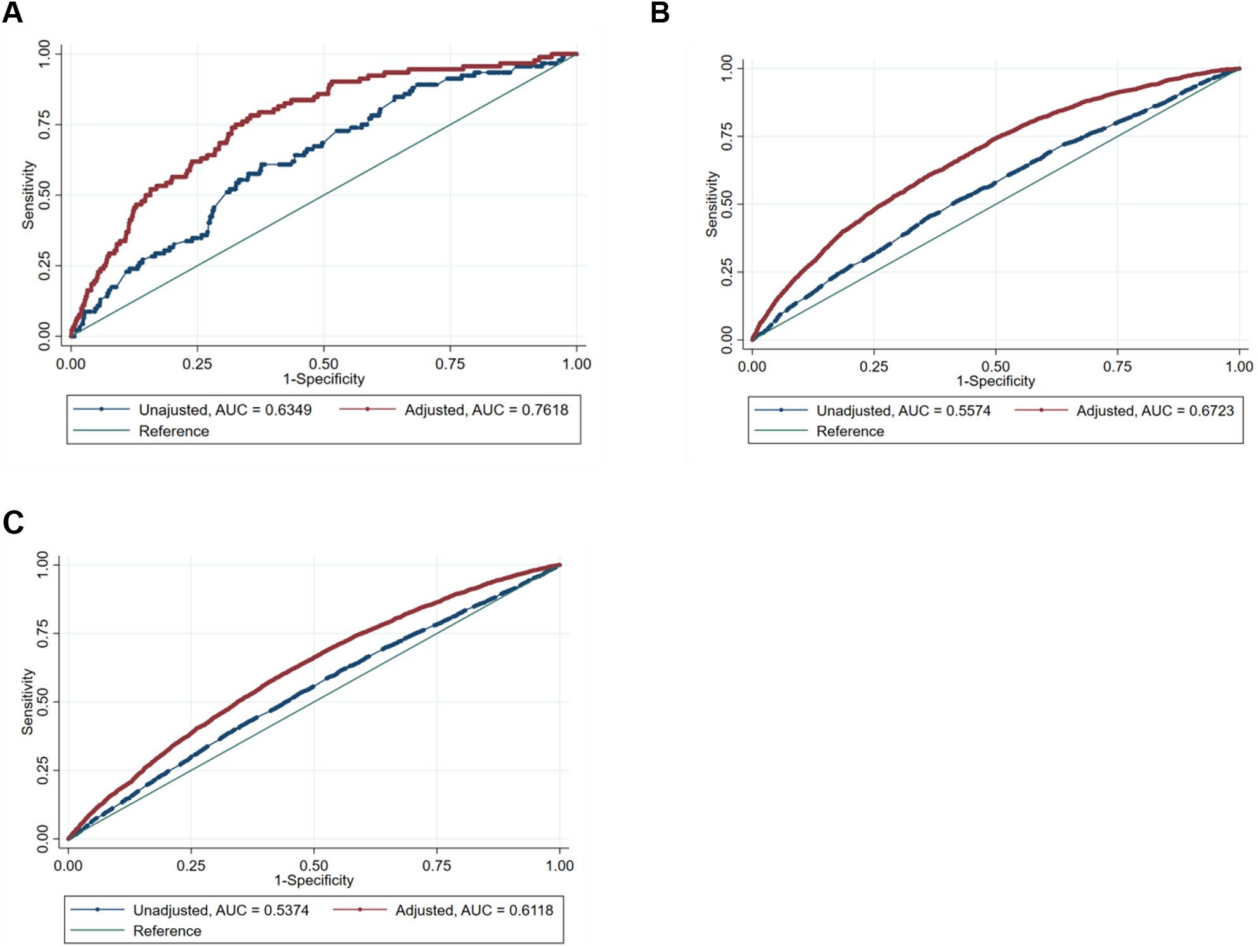
Receiver operating characteristic (ROC) analyses. Figure panel depicts ROC curves based on unadjusted and adjusted logistic regression models for PROMIS depression T-scores in the severe range (Panel **A**), the moderate range (Panel **B**), and the mild range (Panel **C**), versus PROMIS depression symptom T-scores within normal limits (WNL).

ROC curve analyses for each logistic regression model (Fig. 4) showed better discernment based on the adjusted rather than unadjusted models comparing PROMIS depression T-scores between the severe and WNL ranges (AUC = 0.762 vs. unadjusted AUC = 0.635), between the moderate and WNL ranges (AUC = 0.672 vs. unadjusted AUC = 0.557), and between the mild and WNL ranges (AUC = 0.612 vs. unadjusted AUC = 0.537). Using Youden’s Index, which locates the threshold value that maximizes the distance between the ROC curve and the line of chance, to identify optimally performing threshold values from each ROC curve resulted in 85.87% sensitivity to detect PROMIS depression T-scores within the severe range based on the adjusted model, but with specificity of 34.05%; this was the best-performing model in terms of sensitivity. Sensitivity was lowest (42.72%) for detection of PROMIS depression T-scores within the mild range based on an unadjusted analysis. Specificity was lowest (31.55%) for detection of PROMIS depression T-scores within the moderate range based on the adjusted model (Table 4).

**Table 4:** Receiver operating characteristic (ROC) curve analyses for each logistic regression model predicting PROMIS depression symptom T-score categories (mild, moderate, and severe) versus PROMIS depression symptom T-scores within normal limits (WNL) from self-reported body temperature.

Model	Outcome	Temperature Cut point (°C)	Sensitivity	Specificity	ROC AUC
Unadjusted	Severe vs WNL	36.612	60.87%	62.34%	0.635
Adjusted	Severe vs WNL	36.375	85.87%	34.05%	0.762
Unadjusted	Moderate vs WNL	36.624	45.87%	63.40%	0.557
Adjusted	Moderate vs WNL	36.350	75.17%	31.55%	0.672
Unadjusted	Mild vs WNL	36.624	42.72%	63.46%	0.537
Adjusted	Mild vs WNL	36.460	61.76%	44.13%	0.612

*Note*. AUC = Area under the curve; Adjusted = models adjusted for age, biological sex, and self-reported body temperature assessment time of day (see Table 1 note and “Methods”).

### Wearable sensor-assessed temperature

#### Depression symptom frequencies

Of the 21,064 participants with wearable sensor-assessed distal body temperature data, 14,345 had an average depression symptom severity WNL, 4318 had an average depression symptom severity within the mild range, 2330 had an average depression symptom severity within the moderate range, and 71 had an average depression symptom severity within the severe range of the PROMIS depression assessment^28^. The average depression symptom severity score across participants’ mean scores was 50.9 (SD = 2.2), and the mean range between participants’ lowest to highest scores was 5.5 (SD = 5.6), and the median was 4.9 (IQR = 0–8.7).

#### Distributions of wearable sensor-assessed body temperature metrics by depression symptom severity

**Figure 5:**
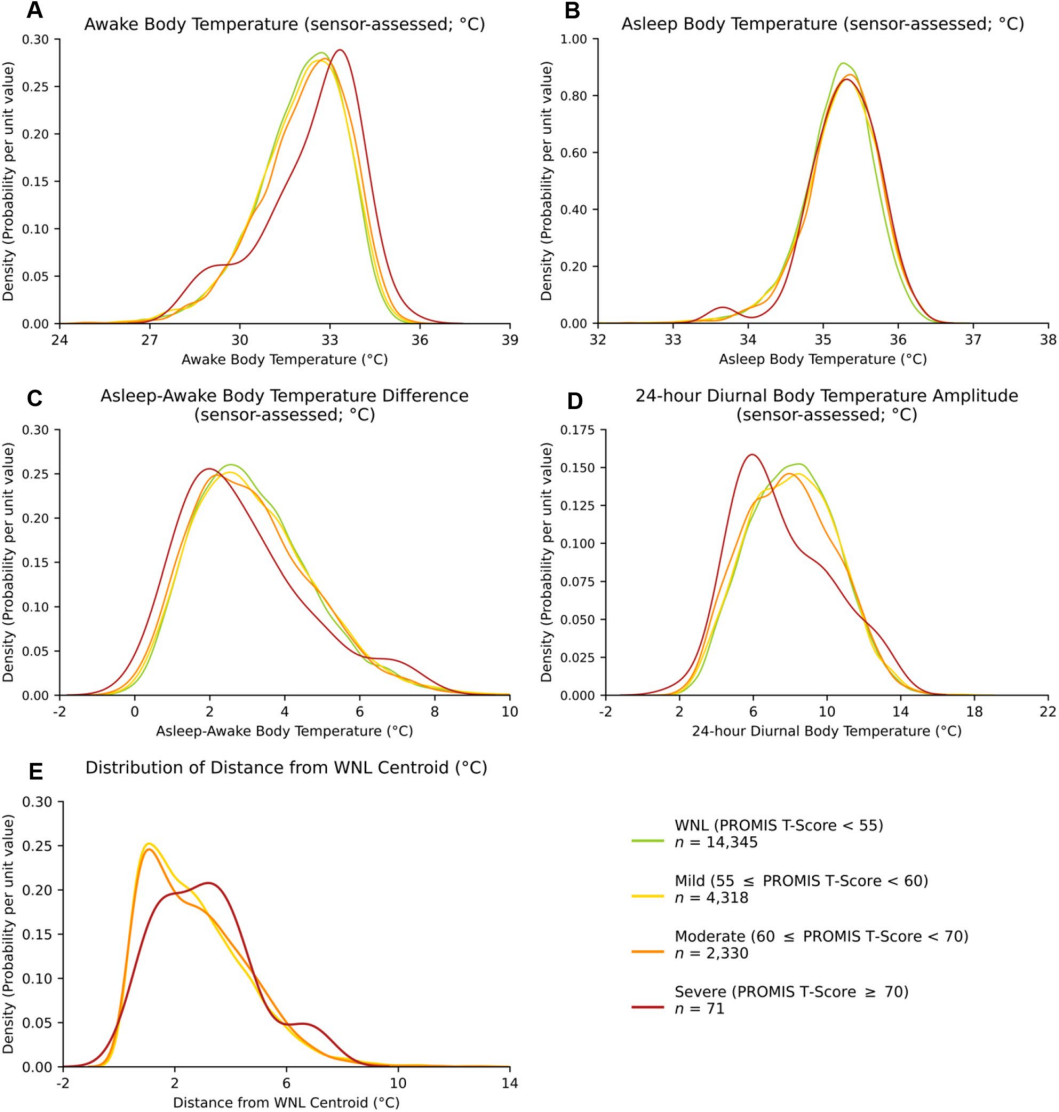
Figure panel depicting wearable sensor-assessed body temperature metrics. Probability Density Plots showing distributions of: Awake distal body temperature (Panel **A**); asleep distal body temperature (Panel **B**); asleep–awake distal body temperature difference (Panel **C**); diurnal distal body temperature amplitude separately for individuals with PROMIS depression T-scores within normal limits (WNL; green), and within the mild (yellow), moderate (orange), and severe (red) PROMIS depression symptom T-score categories (Panel **D**); distribution of the Euclidean Distance (within mild, moderate, and severe depression subgroups) of three distal body temperature metrics (awake temperature, asleep–awake temperature difference, and diurnal distal body temperature amplitude) from the centroid of those metrics among individuals with depression scores WNL (Panel **E**).

Probability density plots illustrate differences in the distribution of 4 wearable sensor-assessed distal body temperature metrics by severity of depressive symptoms (Fig. 5A–D). The awake distal body temperature distributions shift slightly higher from WNL to mild, and from WNL to moderate, with the most pronounced shift from WNL to severe depressive symptoms (see Kolmogorov–Smirnov D-statistics, below). In contrast, the asleep–awake distal body temperature difference and the diurnal distal body temperature amplitude plots demonstrate similar distribution shifts in the other direction; the visible separation of curves largely shows that differences in the mean values for these two variables decrease as depression symptom severity (in comparison to depression symptom scores WNL) increases. The plot for asleep distal body temperature shows more overlap in distributions but some separation of the curves is evident. For diurnal distal body temperatures, the mean within-individual standard deviation was 0.96 for the WNL category, 0.95 for the mild category, 0.91 for the moderate category, and 0.88 for the severe category.

#### Magnitude of associations between wearable sensor-assessed body temperature metrics and depression symptom severity

Kolmogorov–Smirnov D-statistics, which can be considered a measure of effect size, ranged from 0.094 to 0.225 for comparisons of severe symptoms versus WNL for all 4 distal body temperature metrics, while they ranged from 0.014 to 0.056 for comparisons between moderate symptoms versus WNL, and mild symptoms versus WNL (Table 5). Within each of the 4 distal body temperature metrics, the D-statistic was largest for the comparisons of severe symptoms versus WNL and smallest for comparisons of mild symptoms versus WNL. Relative to individuals WNL, individuals with severe depressive symptoms had distal body temperature distributions that were higher during the awake and asleep periods and had lower diurnal amplitudes and smaller asleep–awake temperature differences than individuals with moderate or mild depressive symptoms. The associated statistical tests revealed statistically significant differences between distribution of awake distal body temperature, the asleep–awake difference in distal body temperature, and the diurnal distal body temperature amplitude, but not for asleep distal body temperature, when comparing distributions of these metrics among participants with severe symptoms versus WNL. The distributions of all 4 metrics in participants with moderate symptoms were statistically significantly different from the distributions among participants with symptoms WNL. Only the distribution of asleep distal body temperature in participants with mild symptoms had statistically significant differences compared to participants WNL; comparisons of the distributions of the other 3 metrics between mild symptoms and WNL were not statistically significant.

**Table 5:** Kolmogorov–Smirnov tests comparing wearable sensor-assessed distal body temperature metrics across PROMIS depression symptom T-score categories (severe, moderate, mild) versus PROMIS depression symptom T-scores within normal limits (WNL), with Rank Biserial Correlation (RBC) and its 95% confidence intervals (lower bound, upper bound) for each comparison.

Symptom severity (vs. WNL)	Variable	RBC [95% CI: LB, UB]	D-Statistic	*p*
Severe	Awake distal body temperature	0.173 [0.026, 0.320]	0.225	0.0012
	Asleep distal body temperature	0.088 [–0.044, 0.219]	0.094	0.52
	Asleep–awake distal body temperature difference	–0.129 [–0.270, 0.012]	0.161	0.045
	Diurnal distal body temperature amplitude	–0.158 [–0.303, –0.013]	0.201	0.0053
Moderate	Awake distal body temperature	0.055 [0.029, 0.081]	0.049	1.2 × 10^–4^
	Asleep distal body temperature	0.072 [0.046, 0.097]	0.056	7.8 × 10^–6^
	Asleep–awake distal body temperature difference	–0.028 [–0.053, –0.002]	0.038	0.0067
	Diurnal distal body temperature amplitude	–0.051 [–0.077, –0.025]	0.054	1.6 × 10^–5^
Mild	Awake distal body temperature	0.008 [–0.012, 0.028]	0.014	0.53
	Asleep distal body temperature	0.050 [0.030, 0.070]	0.051	6.7 × 10^–8^
	Asleep–awake distal body temperature difference	0.005 [–0.015, 0.025]	0.017	0.29
	Diurnal distal body temperature amplitude	–0.008 [–0.028, 0.012]	0.020	0.15

*Note*. See Table 2 note.

The Kolomogorov-Smirnov D-statistics shown in Table 5 are a measure of the maximum vertical distance between WNL and all other depression symptom categories on the empirical Cumulative Distribution Function (eCDF) plots (Supplementary Fig. S4). The eCDF plots, as with the density plots in Fig. 5A–D, are another illustration of the shifts in distal body temperature metric distributions with depressive symptom severity. These analyses show that the distal body temperature distributions differed the most between individuals with depressive symptoms in the severe range and individuals with depression symptoms WNL.

The rank biserial correlation coefficients (RBC) comparing each distal body temperature metric for individuals with severe, moderate, or mild depressive symptoms (relative to individuals WNL) showed that the RBC increased as depressive symptoms increased for awake distal body temperature and asleep distal body temperature, consistent with the D-statistics and the increasing mean distal body temperatures with increasing depression symptom severity for these metrics (Table 6). The asleep–awake difference in distal body temperature and diurnal distal body temperature amplitude generally both decreased as symptom severity increased, and correspondingly, the RBC decreased as symptom severity increased.

**Table 6:** Mean values and 95% confidence interval (lower bound, upper bound) of wearable sensor-assessed distal body temperature metrics for each PROMIS depression symptom T-score category.

Variable	PROMIS Depression Symptom T-Score Category Mean 95% CI: [LB, UB]			
	WNL	Mild	Moderate	Severe
Awake distal body temperature	32.038 [32.015, 32.062]	32.051 [32.006, 32.096]	32.166 [32.105, 32.226]	32.366 [31.983, 32.750]
Asleep distal body temperature	35.201 [35.193, 35.209]	35.237 [35.222, 35.253]	35.260 [35.240, 35.279]	35.266 [35.156, 35.377]
Asleep–awake distal body temperature difference	3.163 [3.137, 3.189]	3.186 [3.138, 3.235]	3.094 [3.029, 3.160]	2.900 [2.502, 3.298]
Diurnal distal body temperature amplitude	8.149 [8.110, 8.187]	8.119 [8.046, 8.191]	7.942 [7.842, 8.043]	7.575 [6.958, 8.191]

*Note*. See Table 2 note.

#### Standardized (to WNL) comparisons of body temperature metrics between mild, moderate, and severe depression symptom severities

A probability density plot showing the distribution of the Euclidean Distance of awake distal body temperature, the asleep–awake difference in distal body temperature, and diurnal distal body temperature amplitude to the centroid (mean of these measures among participants WNL), appears in Fig. 5E. This plot shows that all levels of depression symptom severity (mild, moderate, severe) shifted away from the WNL centroid but with much overlap between curves for mild and moderate depressive symptoms, and less overlap with the curve for severe depressive symptoms. This indicates that people with severe depressive symptoms differed more from the WNL values than did people with mild or moderate depressive symptoms. That is, the severe depressive symptoms showed a more pronounced shift away from WNL values compared to the WNL centroid, though this was not statistically significant (Kruskal–Wallis test, *p* = 0.065).

## Discussion

To our knowledge, this is the largest study to date to examine the association between body temperature, assessed using both self-report methods and wearable sensors, and depressive symptoms in a geographically broad sample. In these analyses, higher levels of depressive symptoms were associated with higher body temperatures during time awake. We observed this finding using body temperatures assessed (1) at most once per day via self-collection using handheld thermometers, and (2) at most once per minute via an unobtrusive wearable sensor device (worn on the finger). We found that distal body temperatures collected by the wearable sensor device during sleep were fairly similar across depression categories and were higher than awake distal body temperatures; this resulted in smaller asleep–awake distal body temperature differences with increasing depressive symptom severity. We also found that the association between self-reported body temperature and depressive symptoms was robust to adjusting for time of day at which body temperature was assessed (as done in prior work with smaller samples)^19^. We also observed these associations between self-reported body temperature and depressive symptoms in models that statistically accounted for demographic factors that can affect body temperature^29^.

These findings confirm associations between depressive symptoms and body temperature reported in smaller studies (< 300 participants)^13–16,19–21^. Specifically, these analyses replicated prior results showing that daytime self-reported body temperature was associated with greater depressive symptoms^19,20^ and build on one prior study showing that the asleep–awake body temperature difference was more than twice as large among controls relative to individuals with depression^21^. In contrast to prior work^13,21^; however, we did not observe an association between increased body temperature during sleep time among individuals with greater depressive symptoms. This may be due in part to key differences in measurement; prior studies^13,21^ monitored rectal (core) temperature during the night, whereas the wearable sensor in these analyses was collected from the skin of the finger (distal). As core temperature typically decreases whereas peripheral temperature increases during sleep^30^, it is possible that body temperature increases we observed during sleep are specific to distal temperature. Additionally, these analyses went beyond single self-reported daily body temperature assessments by including minute-level wearable sensor-assessed distal body temperature data over the course of several months, which prior studies of depressive symptoms and body temperature have not done. Importantly, we observed these associations outside of the controlled laboratory setting, which lends further external validity to these findings.

It is uncertain whether the elevated body temperature observed in depression reflects increased metabolic heat production, decreased ability to induce thermoregulatory cooling, or a combination of both. Body temperature reflects a balance between metabolic heat generation and thermoregulatory heat loss, with these processes under tight control by an integrated neural and immune-based feedback system that involves both bodily and central nervous system processes^12^. Available data suggest that the inadequate ability to activate thermoregulatory cooling mechanisms, as indexed by a reduced ability to sweat, may play an important role in the body temperature alterations observed in depression^15,31^. Based on these and other findings, we have previously proposed that MDD may be associated with dysregulation of afferent warm signals from distal cutaneous sensors to the central nervous system, i.e., involving heat-defense mechanisms traveling along the spinoparabrachial pathway^32^, leading secondarily to decreases in sweating as assessed by measurement of skin conductance levels^12,33–36^. Indeed, a recent systematic review and narrative synthesis concluded that lower skin conductance level in individuals with depression versus healthy controls has been a consistent finding^37^.

Evidence suggests that individuals with MDD may have altered electrodermal activity (EDA), which is now the preferred term that encompasses historical terms related to electrical characteristics of the skin, such as electrodermal level, electrodermal response, galvanic skin response, psychogalvanic reflex, skin conductance, skin conductance level, skin conductance response, and sympathetic skin response. EDA is typically assessed as changes in the amount of sweat secreted by eccrine sweat glands in the hypodermis of the palmar region of the hand and plantar region of the foot. EDA has a tonic and a phasic component, with the tonic component being related to skin conductance level), and with the phasic component being related to faster-changing elements of the signal that can be associated with an acute stimulus (skin conductance response) or “spontaneous” or “nonspecific” (nonspecific skin conductance response)^38,39^. Reduced EDA was first associated with depression in 1890^40^, an observation repeatedly observed in depression since then^37,41^. The most consistent findings in individuals with MDD (relative to healthy controls), have been lower skin conductance level, increased skin conductance response latency, and lower skin conductance response amplitude, suggesting alterations of multiple elements of EDA in persons with depression^37^.

Correlations between body temperature metrics and depressive symptoms suggest potential common underlying pathophysiological mechanisms. For example, chronic stressors that contribute to risk for depression^42^ may also impact thermoregulation dysregulation of the hypothalamic pituitary adrenal axis^43–45^. In addition, abnormal glutamate/gamma-aminobutyric acid signaling has been observed in MDD post-mortem brains^46^, and this altered excitatory/inhibitory balance could also contribute to dysregulated body temperature, as shown in models of hot flashes^47^. Finally, low-grade (micro- or para-) inflammation^48^ could lead to both elevated body temperature^49^ and depressive symptoms^50,51^. Interestingly, temperature sensitive channels have been implicated in MDD and bipolar disorder and have also been shown to regulate inflammatory responses^52^ and body temperature^53,54^. Thus, temperature-sensitive immune-regulatory channels are potential targets for development of MDD treatments.

Associations between body temperature and depression might be relegated to the realm of academic interest were it not for data showing that interventions directly targeting thermoregulatory systems have yielded antidepressant effects. Although it may seem counterintuitive that interventions that temporarily raise body temperature could benefit a condition characterized by increased body temperature, acute exposure to high heat induces counter-regulatory thermoregulatory cooling processes that produce longer-term and sustained reductions in body temperature^55^. In the context of MDD, whole-body hyperthermia has been reported to elicit a rapid and sustained reduction in depressive symptoms following a single whole-body hyperthermia treatment designed to raise core body temperature to 38.5 °C^56,57^. Notably, in one of these trials, participants with depression and higher body temperatures prior to WBH tended to experience larger antidepressant responses^57^. Antidepressant effects have also been observed for other heat-based interventions, including hot yoga^58^, hyperthermic baths^59^, and infrared sauna lamps^56,57^. Though these initial studies suggest that alterations in abilities to regulate body temperature may be associated with at least some cases of depression, clarifying the biological pathways through which body temperature is altered in some individuals with depression may reveal more specific pathogenic mechanisms amenable to targeted treatment for individuals with depression and elevated body temperature.

Several limitations of this study warrant discussion and have implications for further investigation. Though most self-reported body temperatures were likely collected using oral assessment methods, participants used personal thermometers to collect their body temperature, and did not report the body site from which they collected their temperature. Unless the site from which participants measured their temperature varied systematically by levels of depressive symptoms, this measurement issue is likely to have made it more difficult to observe an association between body temperature and mood because of increased noise in temperature measurement (thus attenuating observed effects and plausibly rendering them conservative estimates of the true association). Thus, that the reported effects emerged despite this methodological noise is noteworthy. Additionally, we report here the most conservative statistical analysis of difference between groups by aggregating across time to determine average temperature parameters per person. As Fig. 1 makes clear, there are substantial changes for all groups across the year, and we do not attempt to use these changes to reduce the unexplained variance in our comparisons. Given the consistency of the differences visible across the months of data collection, we expect that future classification analyses could generate substantially larger effect sizes than we report here by statistically accounting for time of day, week, month, and year in order to reduce the contribution of these non-random sources of variance from the statistical comparisons.

Although adjusted analyses accounted for the time of day at which participants collected their body temperature, standardizing the time of day at which participants collected their body temperature would have reduced measurement variability. Future research using self-reported body temperature should standardize the thermometer device used, the site from which participants collect their body temperature, and the time of day temperature measurements are collected. Wearable sensor-assessed body temperature may have some additional benefits, such as the provision of continuous assessments that can provide significantly more analytic power. Additionally, wearable sensors may reduce problems associated with human errors in self-collecting body temperature (e.g., improper thermometer placement) and self-reporting (e.g., forgetting to report a value, incorrectly typing in a value).

The wearable sensor-based temperature measurements we used in this study supported the main findings of associations between depressive symptoms and self-reported temperature. Further, they extended these findings, for example, by allowing us to assess associations between depressive symptoms and the difference between awake and asleep body temperature. The wearable sensor devices we used have their own limitations, however. The wearable sensor devices measured dermal temperature on the finger, which can differ from core body temperature. Self-reported body temperatures were likely oral body temperature, which is a metric of core body temperature. The wearable sensor may have artifacts due to measurement errors, for example, due to removing the ring from a finger. To limit measurement artifacts from the wearable sensor, we took steps such as removing the upper and lower 5% body temperature assessments.

We used average depressive symptom scores and body temperature values in these analyses. An important remaining question is whether changes in depressive symptoms are associated with changes in body temperature, and over what timescale. We were unable to assess this well, as depressive symptoms were relatively stable in most of our cohort, with the median range between the highest and lowest depression T-score being 3.9, which may constitute a clinically meaningful difference but relative to categorical depression levels represents a relatively small difference. Although limited prior data suggest that body temperature may decrease when depressive symptoms decrease^21,57^, we were not able to address whether altering body temperature may improve depressive symptoms. As noted, small pilot studies suggest this might be the case^57^, but this is a key area for more rigorous, larger studies in the future. The current analyses reinforce the rationale for such intervention research.

Although we were unable to assess many potential confounders, sensitivity analysis using E-values showed that unmeasured confounders would need to be larger than observed effects of several known relevant variables to explain away all observed effects in these models. Although there are several known risk factors for depression that were not included in this analysis, to function as a confounder, these factors would also need to be associated with elevated body temperature. Genetics play a role in depression, and plausibly may be related to body temperature; however, it seems possible that genetic links to depression and temperature may relate to genetic mediators of depression through a thermoregulatory pathway, rather than constituting unmeasured confounders in observed associations. Illness can play a role in depression and may also influence body temperature, and we were not able to control for this; however, the magnitude of unmeasured potential confounding effects of chronic illnesses causing both depression and elevated body temperature would need to be substantially larger than our observed effects, given the magnitude of the E-values we observed. In these data, ROC curve analysis illustrated the strength of the association between body temperature and depression, with moderate-to-high AUC values across all models, and the highest being for severe depression.

These data confirm an association between body temperature and depressive symptoms in a large sample using body temperature metrics (1) that participants self-collected and self-reported and (2) assessed by wearable sensor devices that automatically transmitted data to the research team, and replicate prior research showing that individuals with depression have smaller asleep–awake body temperature differences. Though depression is biologically, behaviorally, and psychologically heterogeneous, these findings suggest that body temperature may be a candidate biological marker of depression for some individuals with depressive symptoms. Treatments that target elevated body temperature in individuals with both elevated body temperature and depression may warrant further evaluation.

## Methods

### Study design and participants

The TemPredict Study^26,27^ was a prospective, worldwide, cohort study that continuously collected physiological metrics (e.g., body temperature) using an off-the-shelf wearable device (Oura Ring) with the primary aim of developing an algorithm to identify the onset of COVID-19. In addition to data collected by the Oura Ring, participants self-reported their body temperature daily and completed monthly surveys. Average study engagement was 127.1 days for the self-reported body temperature analyses and 87.7 days for the sensor-assessed distal body temperature analyses. Eligible participants were at least 18 years of age, possessed a smartphone that could pair with the Oura Ring, and could communicate in English. Eligible participants either already possessed an Oura Ring that they used for participation or were provided with an Oura Ring for participation (the study provided Oura Rings to frontline healthcare workers at several participating healthcare institutions). We conducted participant recruitment through invitations delivered within the Oura smartphone app; these invitations contained embedded weblinks for prospective participants to review study details and to begin the study enrollment process^60^. All participants provided electronic informed consent. Participants provided consent to use data from their personally owned Oura Ring prior to their enrollment; we therefore analyzed data collected by the wearable devices beginning in April 2020. We did not pay participants for participation. The University of California, San Francisco, Institutional Review Board (IRB; IRB# 20-30408) and U.S. Department of Defense, Human Research Protection Office (HRPO; HRPO# E01877.1a) approved of all study procedures, and all research was performed in accordance with relevant guidelines and regulations and the Declaration of Helsinki. We followed the Strengthening the Reporting of Observational Studies in Epidemiology (STROBE) reporting guidelines^61^.

### Measures

#### Depression severity

Participants received a monthly survey (Qualtrics Survey Software) via email that included the Patient Reported Outcomes Medical Information System (PROMIS) Adult Health Profile instrument for depression (v1.0 Form 4a) with the recall period modified (as described elsewhere^62^) to reflect the past month rather than the past 7 days^63^.

#### Self-reported body temperature

Participants self-reported their demographic information, including age and biological sex, in a baseline survey. Participants self-reported their self-assessed body temperature once per day by accessing a daily self-reported survey (Qualtrics Survey Software) that was linked within the Oura App. The daily survey asked participants to report the highest body temperature value they collected within the last day (in °F or °C, per participant preference). For these daily self-reported temperature assessments, participants used their own thermometers (which were not provided by the study, and which may have been of any brand), and we did not ask participants to report the type of thermometer used or the method of body temperature assessment. Qualtrics generated a time stamp for each completed survey.

#### Wearable sensor-assessed body temperature

All participants wore the Oura Ring Gen2 (ouraring.com), a commercially available wearable sensor device (Oura Health, Oulu, Finland), on a finger of their choosing, as previously described^27^. The Oura Ring connects to the Oura App (available in Google Play Store and Apple App Stores) via Bluetooth. Users can wear the ring continuously during daily activities and sleep in wet and dry environments. The Oura Ring assesses distal body temperature using a negative temperature coefficient (NTC) thermistor (resolution of 0.07 °C) on the internal surface of the ring, which is in relatively consistent contact with the skin. The wearable sensor registers distal (dermal, peripheral) body temperature readings from the palm side of the finger base every minute. As described elsewhere^27,64^, the Oura Ring also assesses other physiological metrics, including sleep parameters, heart rate, heart rate variability, respiratory rate, and activity metrics.

### Data analyses

We performed statistical analyses using Stata v16 and Python v3.8.10 with the *statsmodels* (v0.13.2), *scipy* (v1.8.0), and *scikit_posthocs* (v0.8.0) packages.

#### Variable preparation

##### Depression severity

Each item on the PROMIS depression Form 4a is scored from 1 (*never*) to 5 (*always*), and these item scores are summed to create a total raw summary score. We converted raw PROMIS depression summary scores to T-scores using the conversion tables in the PROMIS scoring manual (healthmeasures.net^65^). PROMIS T-scores are referenced to a general adult population with a mean score of 50 and standard deviation of 10, with higher T-scores indicating greater depression symptomatology^63,66^.

##### Self-report body temperature

We excluded self-reported body temperature data points that were above 38.0 °C, as these are not representative of baseline temperature and meet criteria for clinical fever. We excluded these periods of potential illness because the focus of this analysis was body temperature under usual conditions of daily life, and so we excluded known periods of aberrant physiology. We used the Qualtrics auto-generated timestamp to create self-reported body temperature “time of day variables” as done in prior work examining associations between oral temperature and self-reported depressive symptoms^19^. Specifically, we calculated the cosine (B1) and sine (B2) of 2*pi*t for each self-reported body temperature value, where “t” was the decimal proportion of the day during which the daily survey was completed.

##### Wearable sensor-assessed distal body temperature

For each participant, we used the two weeks of distal body temperature data prior to each completed PROMIS assessment. For each 24-h period, we discarded wearable sensor-assessed distal body temperature values below the 5th percentile value and above the 95th percentile. This served to exclude periods of non-wear and periods of clinical fever and to minimize influence from non-representative extreme values (e.g., outliers), as done in prior research with wearable sensor-assessed metrics^67–69^. We used hypnogram data to determine periods of asleep versus awake. As done in prior studies of body temperature and depression^19,21^, we computed metrics for the daily average distal body temperature during minutes awake (“awake distal body temperature”), the daily average during sleeping minutes (“asleep distal body temperature”), and the difference between those averages (“asleep–awake distal body temperature difference”). To compute the diurnal distal body temperature amplitude, we calculated the daily maximum distal body temperature as the highest daily value and the daily minimum distal body temperature as the lowest daily value for each person and calculated the difference between these two values.

#### Statistical approach

##### Self-report body temperature

Analyses of self-reported body temperature data included participants who completed one or more PROMIS depression assessments and who provided at least one self-reported temperature assessment, resulting in a sample of 20,880 participants.

We computed an unadjusted linear regression model with participants’ average self-reported body temperature as the predictor variable and average PROMIS depression T-score as the outcome variable. We also computed a multiple linear regression model that adjusted for age and biological sex, both of which can affect body temperature^29^, as well as body temperature survey time stamp (as done in prior analyses of depression and body temperature^19^). We included each participant’s body temperature time-of-day variables (average B1 and B2) in adjusted models to hold constant the average effect of time of day for each participant’s average body temperature. We plotted body temperatures for each depression T-score category.

We then conducted unadjusted and adjusted logistic regression models with categorical depression level variables (mild, moderate, and severe, each with WNL as the reference category) as outcomes. We used established thresholds for PROMIS depression T-scores, with separate models for mild (T-score 55–59.99) vs. WNL (T-score below 55); moderate (T-score 60–69.99) vs. WNL; and severe (T-score 70 or greater) vs. WNL, as the outcome variable, and self-reported body temperature as the predictor, scaled per 0.1 °C^63^. We report odds ratios with 95% confidence intervals (CIs) for the odds for each depression level with a 0.1 °C increase in average self-reported body temperature and visualized this in a forest plot. For each linear and logistic regression model, we performed sensitivity analyses by calculating E-values^70,71^, which provide estimates of the magnitude of unmeasured confounding that could explain the observed effects and have been recommended as a type of sensitivity analysis^70,72^. We conducted receiver operating characteristics (ROC) curve analyses for each logistic regression model and calculated the area under the curve (AUC) to evaluate how well self-reported body temperature could identify depressive symptoms in three depression comparisons (WNL vs. mild; WNL vs. moderate, and WNL vs. severe), and used Youden’s Index to identify body temperature thresholds to evaluate sensitivity and specificity based on those threshold values^73^. Youden’s index can be calculated as (sensitivity + specificity –1) and ranges between 1 and 0, with 1 indicating perfect sensitivity and specificity and 0 indicating results no better than chance^73^. Maximizing Youden’s index in ROC analysis identifies the value of a continuous predictor that best distinguishes cases and non-cases, and is equivalent to locating the point on the curve that is furthest from the line of chance^74^. To control for multiple comparisons, we set the significance level at *p* < 0.01 for all statistical tests (all two-sided).

##### Wearable sensor-assessed body temperature

Analyses involving wearable sensor-assessed distal body temperature data included participants who completed one or more PROMIS depression assessments and wore an Oura Ring for at least seven 24-h periods with at least 4 h of asleep and awake distal body temperature data recorded during each 24-h period, resulting in a sample of 21,064 participants. We included distal body temperature data collected in the 2 weeks prior to each completed PROMIS depression assessment.

We grouped participants into WNL, mild, moderate, or severe depression PROMIS depression symptom categories^75,76^. We plotted probability density plots to illustrate differences in the distributions of the four distal body temperature metrics by severity of depressive symptoms. We then calculated Kolmogorov–Smirnov Distance (D-statistic) and *p*-values, together with rank biserial correlations and their corresponding 95% confidence intervals, to quantify the magnitude of the association between distal body temperature metrics and depression symptom severity. We also created empirical Cumulative Distribution Function (eCDF) plots. Briefly, the Kolmogorov–Smirnov D-statistic is proportional to the maximum vertical distance between two eCDF plots, and ranges from 0 (distributions entirely overlapping) to 1 (distributions completely separated), functioning as a relative effect size metric for the comparison between two empirical distributions^77,78^. Together, the above analyses allowed us to compare the distributions of distal body temperature metrics of the three depression symptom categories (mild, moderate, severe) relative to WNL. We also calculated Rank Biserial Correlations^79,80^ with 95% confidence intervals^81^. To further explore distal body temperature differences between depression symptom categories, we computed the standardized distal body temperature metrics within each level of depression symptom severity (mild, moderate, and severe) against those from the WNL centroid (the Euclidean Distance). We calculated the WNL centroid as mean of awake distal body temperature, asleep–awake temperature difference in distal body temperature, and diurnal distal body temperature amplitude; we did not include asleep distal body temperature within this centroid calculation as asleep distal body temperature did not diverge based on depression symptom category. We calculated the Euclidean Distance as the distance between the WNL centroid and each participants’ values on each of three distal body temperature metrics (awake distal body temperature, asleep–awake temperature difference in distal body temperature, and diurnal distal body temperature amplitude). We then created density plots for the distribution of the Euclidean Distance; we did this within each depression category (mild, moderate, severe) and computed a Kruskal–Wallis test.

### Supplementary Information

Supplementary Information.

## Data Availability

Oura’s data use policy does not permit us to make wearable sensor-assessed (via the Oura Ring) data available to third parties. Self-report data (e.g., self-reported body temperature, self-reported depressive symptoms) can be made available; those seeking to reproduce our findings should contact Ashley Mason, PhD, and Benjamin Smarr, PhD for an online application to access the study data portal. This application process will require requesters to make a written commitment expressing agreements to not duplicate data, to not share data with third parties, and/or other confidentiality precautions.
