# Author Correction: Elevated body temperature is associated with depressive symptoms: results from the TemPredict Study

**DOI:** 10.1038/s41598-024-60565-x

**Published:** 2024-04-29

**Authors:** Ashley E. Mason, Patrick Kasl, Severine Soltani, Abigail Green, Wendy Hartogensis, Stephan Dilchert, Anoushka Chowdhary, Leena S. Pandya, Chelsea J. Siwik, Simmie L. Foster, Maren Nyer, Christopher A. Lowry, Charles L. Raison, Frederick M. Hecht, Benjamin L. Smarr

**Affiliations:** 1 Osher Center for Integrative Health, University of California San Francisco, San Francisco, CA USA; 2 Shu Chien-Gene Lay Department of Bioengineering, University of California San Diego, San Diego, CA USA; 3 Neurosciences Graduate Program, University of California San Diego, San Diego, CA USA; 4 Department of Management, Zicklin School of Business, Baruch College, The City University of New York, New York, NY USA; 5 Department of Psychology, The University of Arizona, Tucson, AZ USA; 6 Department of Wellness and Preventative Medicine, Cleveland Clinic, Cleveland, OH USA; 7 Depression Clinical and Research Program, Massachusetts General Hospital, Boston, MA USA; 8 Department of Psychiatry, Harvard Medical School, Boston, MA USA; 9 Department of Integrative Physiology, University of Colorado Boulder, Boulder, CO USA; 10 Department of Psychiatry, School of Medicine and Public Health, University of Wisconsin-Madison, Madison, WI USA; 11 Halıcıoğlu Data Science Institute, University of California San Diego, San Diego, CA USA

Correction to: *Scientific Reports* 10.1038/s41598-024-51567-w, published online 05 February 2024

The original version of this Article contained errors in Table 4, results for the receiver operating characteristic curve analyses.

Table 4, “Receiver operating characteristic (ROC) curve analyses for each logistic regression model predicting PROMIS depression symptom T-score categories (mild, moderate, and severe) versus PROMIS depression symptom T-scores within normal limits (WNL) from self-reported body temperature.”

**Table:** 

Model	Outcome	Temperature Cut point (°C)	Sensitivity	Specificity	ROC AUC
Unadjusted	Severe vs WNL	36.613	60.87%	62.34%	0.635
Adjusted	Severe vs WNL	36.375	85.87%	34.05%	0.762
Unadjusted	Moderate vs WNL	36.624	96.91%	63.40%	0.557
Adjusted	Moderate vs WNL	36.350	75.17%	31.55%	0.672
Unadjusted	Mild vs WNL	36.624	42.72%	63.46%	0.537
Adjusted	Mild vs WNL	36.500	58.54%	47.47%	0.612

now reads,

**Table:** 

Model	Outcome	Temperature Cut point (°C)	Sensitivity	Specificity	ROC AUC
Unadjusted	Severe vs WNL	36.612	60.87%	62.34%	0.635
Adjusted	Severe vs WNL	36.375	85.87%	34.05%	0.762
Unadjusted	Moderate vs WNL	36.624	45.87%	63.40%	0.557
Adjusted	Moderate vs WNL	36.350	75.17%	31.55%	0.672
Unadjusted	Mild vs WNL	36.624	42.72%	63.46%	0.537
Adjusted	Mild vs WNL	36.460	61.76%	44.13%	0.612

Additionally, in the Results section, ‘Receiver operating characteristics (ROC) curve analyses’

“Using Youden’s Index, which locates the threshold value that maximizes the distance between the ROC curve and the line of chance, to identify optimally performing threshold values from each ROC curve resulted in 85.87% sensitivity to detect PROMIS depression T-scores within the severe range based on the adjusted model, but with specificity of 34.05%; the best performance was 96.91% sensitivity to detect PROMIS depression T-scores within the moderate range (with 63.40% specificity) from the unadjusted model. Sensitivity was lowest (42.72%) for detection of PROMIS depression T-scores within the mild range based on an unadjusted analysis. Specificity was lowest (31.55%) for detection of PROMIS depression T-scores within the moderate range based on the adjusted model (Table 4).”

now reads,

“Using Youden’s Index, which locates the threshold value that maximizes the distance between the ROC curve and the line of chance, to identify optimally performing threshold values from each ROC curve resulted in 85.87% sensitivity to detect PROMIS depression T-scores within the severe range based on the adjusted model, but with specificity of 34.05%; this was the best-performing model in terms of sensitivity. Sensitivity was lowest (42.72%) for detection of PROMIS depression T-scores within the mild range based on an unadjusted analysis. Specificity was lowest (31.55%) for detection of PROMIS depression T-scores within the moderate range based on the adjusted model (Table 4).”

The original version of this Article has been corrected.

