# Supplementary material for: Elevated body temperature is associated with depressive symptoms: results from the TemPredict Study
Source: Sci Rep. 2024 Feb 5;14:1884. doi: 10.1038/s41598-024-51567-w (PMC10844227; doi:10.1038/s41598-024-51567-w)
Supplement: Supplementary file 1 — Supplementary Information. [file 41598_2024_51567_MOESM1_ESM.docx]

**Supplementary Table 1.** Standardized linear regression model regressing average PROMIS depression symptom T-scores onto average self-reported body temperature.

| **Model** | ***b*** | **SE** | ***P*** | **95% CI [LB, UB]** |
| --- | --- | --- | --- | --- |
| Adjusted Model (*n* = 20,863), *r*^2^ = 0.078 |  |  | 2.3x10^-128^ |  |
| Self-report body temperature | 0.0458 | 0.0067 | 9.5x10^-12^ | [0.0327, 0.0590] |
| Age | -0.1761 | 0.0066 | 1.0x10^-154^ | [-0.1892, -0.1631] |
| Biological sex | -0.1856 | 0.0065 | 3.7x10^-177^ | [-0.1984, -0.1728] |
| Time of day (B1) | -0.0316 | 0.0070 | 7.3x10^-6^ | [-0.0454, -0.0178] |
| Time of day (B2) | -0.0581 | 0.0071 | 3.7x10^-16^ | [-0.0721, -0.0441] |

*Note.* See Table 1 note.

**Supplementary Figure 1.** Participant flow diagram for self-reported body temperature analyses. Diagram depicts self-reported body temperature analytic sample selection.

*Note***.** *Number updated since publication of primary outcomes^27^; we were able to add 1,345 previously excluded participants who had changed their Oura App identifier by further working to match updated Oura App identifiers with study identification numbers.

**Supplementary Figure 2.** Participant flow diagram for wearable sensor-assessed body temperature analyses. Diagram depicts wearable sensor-assessed body temperature analytic sample selection.

*Note***.** See Supplementary Figure 1 note.

**Supplementary Figure 3. Average self-reported body temperature (A) and average wearable sensor-assessed distal body temperature (B) plotted by PROMIS depression symptom T-score categories (unsmoothed)**. Figure panels show that individuals with PROMIS depression symptom T-scores within normal limits (WNL; green) have the lowest average self-reported and wearable sensor-assessed distal body temperatures, with increasing average self-reported and wearable sensor-assessed distal body temperatures among individuals in the mild (yellow), moderate (orange), and severe (red) PROMIS depression symptom T-score categories.


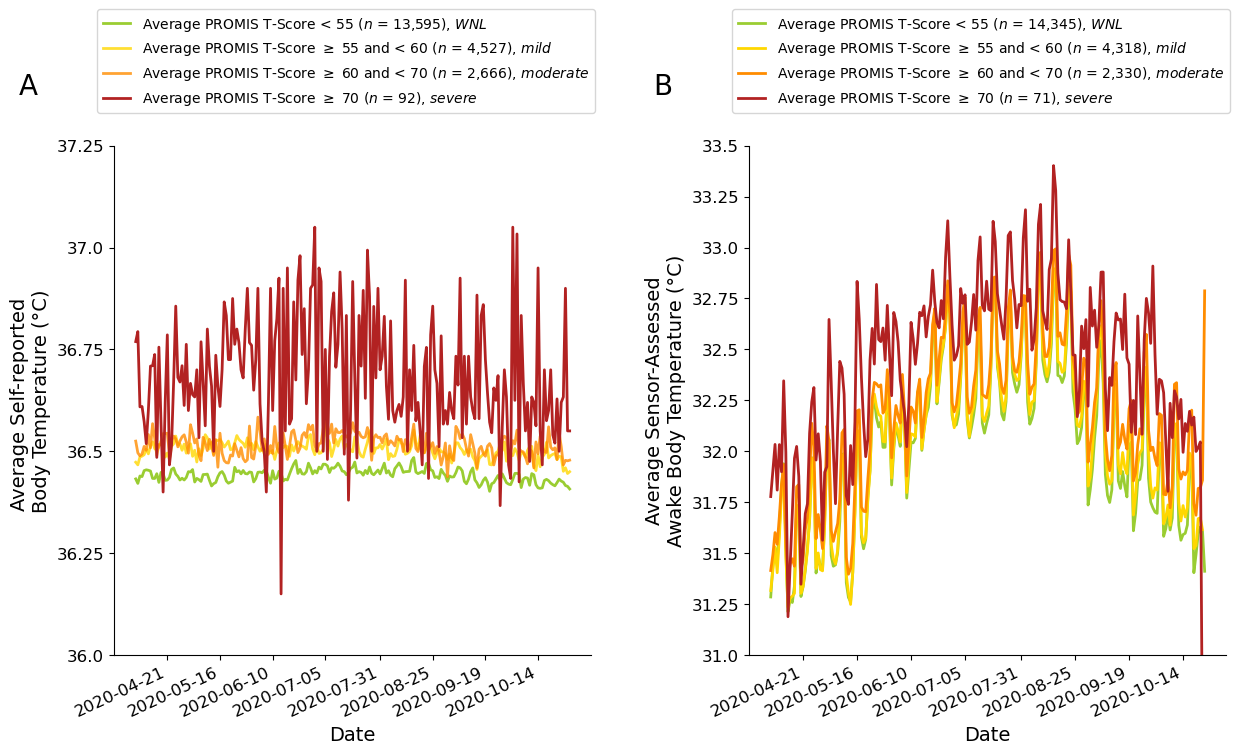


*Note***.** Average self-reported and wearable sensor-assessed body temperatures (°C), unsmoothed. See Figure 1 for smoothed comparators.

**Supplementary Figure 4.** Empirical cumulative distribution function of sensor-assessed distal body temperature metrics. Awake distal body temperature (Panel A), asleep distal body temperature (Panel B), asleep-awake distal body temperature difference (Panel C), and diurnal body temperature amplitude (Panel D) separately for individuals with PROMIS depression T-scores within normal limits (WNL; green), and within the mild (yellow), moderate (orange), and severe (red) PROMIS depression symptom T-score categories.

**
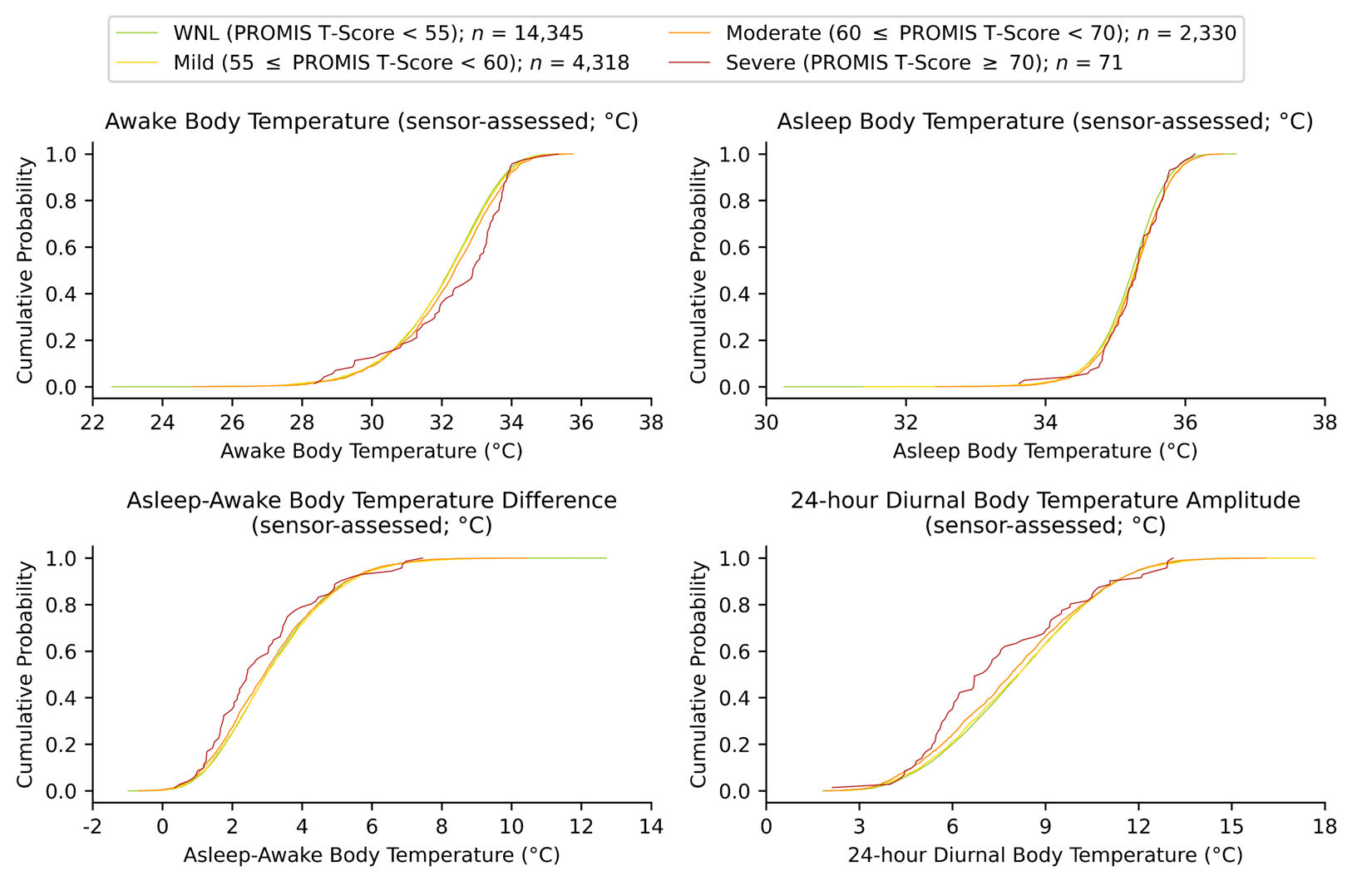
**

**B**

**A**

**D**

**C**
